# Genome-wide transcription factor binding site/promoter databases for the analysis of gene sets and co-occurrence of transcription factor binding motifs

**DOI:** 10.1186/1471-2164-11-145

**Published:** 2010-03-01

**Authors:** Srinivas Veerla, Markus Ringnér, Mattias Höglund

**Affiliations:** 1 Department of Oncology, Clinical Sciences, Lund University and Lund University Hospital, SE-22185 LUND, Sweden; 2 Computational Biology and Biological Physics, Department of Theoretical Physics, Lund University, SE-22362 LUND, Sweden

## Abstract

**Background:**

The use of global gene expression profiling is a well established approach to understand biological processes. One of the major goals of these investigations is to identify sets of genes with similar expression patterns. Such gene signatures may be very informative and reveal new aspects of particular biological processes. A logical and systematic next step is to reduce the identified gene signatures to the regulatory components that induce the relevant gene expression changes. A central issue in this context is to identify transcription factors, or transcription factor binding sites (TFBS), likely to be of importance for the expression of the gene signatures.

**Results:**

We develop a strategy that efficiently produces TFBS/promoter databases based on user-defined criteria. The resulting databases constitute all genes in the Santa Cruz database and the positions for all TFBS provided by the user as position weight matrices. These databases are then used for two purposes, to identify significant TFBS in the promoters in sets of genes and to identify clusters of co-occurring TFBS. We use two criteria for significance, significantly enriched TFBS in terms of total number of binding sites for the promoters, and significantly present TFBS in terms of the fraction of promoters with binding sites. Significant TFBS are identified by a re-sampling procedure in which the query gene set is compared with typically 10^5 ^gene lists of similar size randomly drawn from the TFBS/promoter database. We apply this strategy to a large number of published ChIP-Chip data sets and show that the proposed approach faithfully reproduces ChIP-Chip results. The strategy also identifies relevant TFBS when analyzing gene signatures obtained from the MSigDB database. In addition, we show that several TFBS are highly correlated and that co-occurring TFBS define functionally related sets of genes.

**Conclusions:**

The presented approach of promoter analysis faithfully reproduces the results from several ChIP-Chip and MigDB derived gene sets and hence may prove to be an important method in the analysis of gene signatures obtained through ChIP-Chip or global gene expression experiments. We show that TFBS are organized in clusters of co-occurring TFBS that together define highly coherent sets of genes.

## Background

The use of global gene expression profiling is a well established approach to characterize biological states or responses. One of the major goals of these investigations is to identify sets of genes with similar expression patterns that may shed new light on the underlying biological process leading to the observed states. A logical and systematic next step is to reduce the identified gene signatures to the regulatory components that induce the relevant gene expression states, with the over all goal to identify the gene regulatory network in operation. A central issue in this context is to identify transcription factors, or transcription factor binding sites, likely to be of importance for the expression of the gene signatures. A constructive approach in this direction has been to combine information on specific transcription factor binding sites (TFBS) with information on gene co-expression as determined by global gene expression analysis. The first step in this effort is to identify putative TFBSs in a set of gene promoters. This is normally accomplished by searching the DNA sequence for matches to generalized sequence patterns obtained from experimentally characterized binding sites. These sequence patterns are represented in the form of position weight matrices (PWM) that describe the probability distribution of the four possible nucleotides at each location in the motif sequence. Several softwares such as MATCH [1], MatInspector [2], and TESS [3] use PWMs obtained from JASPAR [4] or TRANSFAC [5] - large and frequently updated databases that contain PWMs - to identify possible binding sites. An inherent problem with TFBS searches is that the binding motifs are short and degenerate which lead to high error rates in genome-wide scans. One approach to reduce the error rate is to limit the search to evolutionarily conserved segments or to evolutionarily conserved binding sites only. In addition, when working with sets of coordinated genes the search may be restricted to TFBS shared by all members in the gene set, or show over-representation. Hence, a productive method has been to select genes that are significantly changed or show coordinated expression, and then identify over-represented TFBSs in the promoters of the selected genes. For example, in the TOUCAN software [6] Markov background models are used to identify significant binding sites analytically and a parametric statistical algorithm to estimate enrichment, whereas in the CONFAC software [7] evolutionarily conserved binding sites are considered significant and TFBS enrichment is evaluated by a non-parametric statistical test using a fixed set of reference random gene lists. An alternative approach to include gene expression analyses in the protocol is to produce gene sets based on shared TFBS and investigate the extent to which genes in the sets show co-expression in published gene expression data [8]. An extension of this approach is to score all genes for high quality TFBS and then investigate to what extent TFBS co-occur with the aim to identify genes that show a high probability to be regulated by the same sets of transcription factors. In these latter approaches efficient means to produce lists of possible target genes is necessary. In the present investigation we develop a strategy that efficiently produces TFBS/promoter databases. The resulting TFBS/promoter databases constitute all genes in the Santa Cruz database and the positions for all TFBS provided by the user as PWMs. These databases are then used for two purposes, to identify significant TFBS in the promoters of gene sets and to identify clusters of co-occurring TFBS on a genome-wide scale. In the first application significant TFBS are identified by a re-sampling procedure in which the query gene set is compared with typically 10^5 ^independent gene lists of similar size randomly drawn from the TFBS/promoter database. For these purposes two criteria for significance are used; significant enrichment and significant presence. We apply this strategy to both published ChIP-Chip data and to curated gene signatures obtained from the MSigDB database. In the second application, we show that several groups of TFBS are highly correlated and that correlated TFBS define functionally related sets of genes.

## Results

### Creating TFBS/promoter databases

To generate TFBS/promoter databases we developed the SMART (Systematic Motif Analysis Retrieval Tool) software. The SMART software contains an application to download RefSeqs, mRNA information, and promoter regions from the UCSC Genome browser database to a local MySQL database, and hence reduce the need for frequent interactions with the UCSC Genome browser database. The SMART software then retrieves each promoter sequence from the local database followed by scanning for transcription factor binding sites. In this study we used PWMs from the TRANSFAC v2009.1 database [5]. For the first TFBS/promoter database, core similarity score (CSS) threshold values of 1.0 and matrix similarity score (MSS) threshold values of 0.9, were used to identify putative TFBS [1]. All hits of TFBS for the same transcription factor, e.g., MYC_01, MYC_02, and MYC_03, in a given promoter region were then summed up. A first TFBS/promoter database was produced using promoter sequences consisting of the - 1500 to +500 genomic interval relative putative TSSs, using the start of the RefSeq or the mRNA with the most distal TSS for each gene. Then a second TFBS/promoter database aimed for phylogenic foot printing analyses was created by using the corresponding orthologous genes listed in the NCBI HomoloGene database based on human and mouse. For each orthologous gene pair the evolutionarily conserved sequences within the - 5000 to +1000 bp relative the TSS were identified using the BLAST program and the criterion e < 0.001, and TFBS identified as above. In the present investigation the -1500 to +500 bp TFBS/promoter database is composed of 18 377 genes and the - 5000 to +1000 bp database of 13 117 genes.

### Estimations of significance using TFBS/promoter databases as references

We use two measures of significance to identify TFBS likely to be involved in the regulation of the query gene sets; significant enrichment (E); an estimation of TFBS over-abundance in a query gene list, and significant presence (P); an estimation of TFBS promoter over-presence in a query gene list. TFBS enrichment analysis is based on the observation that genes regulated by a given transcription factor tend to have several TFBS for the factor in their respective promoter regions. To calculate E the total number of instances of a given TFBS in the promoters of genes in a query gene list is recorded. Then the TFBS/promoter database is re-sampled with replacement to produce randomly selected and independent gene lists with the same number of genes as in the query list. The number of TFBS instances in each re-sampled gene list is recorded and then ranked according to the number of TFBS present (the gene list with the largest number of instances = rank 1) together with the results for the query list. The rank of the query gene list divided by the total number of re-sample instances is then used as a p value for the probability to acquire the level of enrichment by chance (P_E_). The P_E _value is then transformed into a score by using the -Log(P_E_) value. Assuming that all genes in a given query list are regulated by a given transcription factor TF_A_, one would expect that each gene promoter contains at least one binding site for TF_A_. The chance to find one instance of a given TFBS in a promoter is however dependent on the frequency of the corresponding sequence motif in promoters. We therefore introduce the "significant presence" measure which is an estimate of the probability (P_P_) to find the observed number of promoters with at least one instance of the TFBS in a randomly selected gene list of the same length as the query list. The P_P _is estimated by a resampling procedure similar to the estimation of P_E_, and is converted to a score by using -Log(P_P_). The fraction of genes (%-presence) in the query list that contain at least one instance of a given TFBS is also recorded.

### Gene set promoter analysis

**Figure 1:**
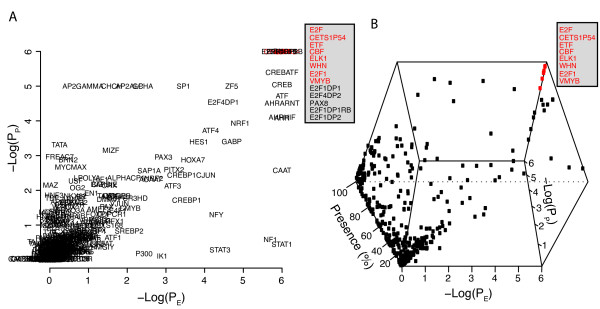
**Promoter analysis of genes binding E2F4 as determined by ChIP-Chip analysis**. Estimations of P_E _and P_P _based on 10^6 ^re-samples and the -1500,+500 portions of the promoters. A) TFBS E-score plotted against P-score. Insert; list of binding sites with maximum -Log(P_E_) and -Log(P_P_), motifs in red; motifs with ≥ 80% presence, B) a three dimensional representation of the results with E-score on the x-axis, P-score on the y-axis, and %-presence on the z-axis. Insert, motifs with -Log (P_E_) and -Log(P_P_) equal to 6 and showing ≥ 80% presence.

To validate the promoter analysis strategy it was first applied to published ChIP-Chip data and then curated gene sets from MSigDB. The rationale of using ChIP-Chip data is that such data is expected to identify gene promoters that a given transcription factors interact with physically. The results are either evaluated by a table containing values for P_E_, P_P_, and %-presence, respectively, or by score graphs produced by plotting the -Log(P_E_), -Log(P_P_), and %-presence, in any combination. An example of a -Log(P_E_)/-Log(P_P_) graph is given in Figure 1A in which the promoters of genes reported by Boyer et al. to bind E2F4 by ChIP-Chip analysis (BOYER_E2F4) have been analyzed using the -1500 to +500 bp surrounding the TSS's and a total of 10^6 ^re-samples. The analysis identified the TFBS E2F, E2F1, E2F1DP1, E2F4DP2, E2F, E2F1DP1RB and E2F1DP2, among others, as both significantly enriched and significantly present with E and P scores of 6 in each case (Table 1). In Figure 1B a three dimensional representation of the data is shown based on the -Log(P_E_) and -Log(P_P_) scores, and %-presence. This latter graph identifies E2F, E2F1, CBF, ETF, WHN, VMYB, ELK1, and CETS1P54 as highly significant binding sites.

**Table 1:** TFBS E and P scores for the BOYER_E2F4 data

TFBS	E Score	P score	%-presence
E2F	6.00	6.00	97.1
CETS1P54	6.00	6.00	96.7
ETF	6.00	6.00	96.7
CBF	6.00	6.00	93.6
ELK1	6.00	6.00	92.0
WHN	6.00	6.00	92.0
E2F1	6.00	6.00	87.6
VMYB	6.00	6.00	79.9
E2F1DP1	6.00	6.00	57.7
E2F4DP2	6.00	6.00	57.7
PAX8	6.00	6.00	52.9
E2F1DP1RB	6.00	6.00	40.5
E2F1DP2	6.00	6.00	28.3
CREBATF	6.00	5.05	77.0
CREB	6.00	5.00	77.2
ATF	6.00	4.77	76.5
AHRARNT	6.00	4.55	84.1
AHR	6.00	4.15	92.9
AHRHIF	6.00	4.07	92.9
ZF5	4.07	6.00	90.3
SP1	3.57	6.00	96.2
CAAT	6.00	2.59	67.3
GC	2.23	6.00	80.8
AP2ALPHA	2.20	6.00	94.9
CHCH	1.60	6.00	97.6
AP2GAMMA	0.86	6.00	85.8
NF1	5.30	0.58	98.0
STAT1	6.00	0.45	100.0

Apart from data on E2F4 binding sites Boyer et al. also reported ChIP-Chip data on NANOG, OCT4, and SOX2 binding sites. As no definite PWM exists for SOX2 in the TRANSFAC database no analyses of this binding site were performed. The analyses of the genes reported to bind NANOG or OCT4 using -1500 to +500 portions of the promoters did not indicate any binding sites for the NANOG and OCT related transcription factors as significant. We then applied a phylogenic foot printing approach using the -5000,+1000 portions of the promoters (Additional file 1, Figure S1A and B). In this case, NANOG and OCT related binding sites were among the top ranking binding sites with respect to E- and P-scores in the BOYER_NANOG and the BOYER_OCT4 data sets respectively. NANOG showed E- and P-scores of 4.7 and 5, respectively, and the OCT related binding sites OCT4, OCT1, and OCT all showed maximum E- and P-scores of 5 when using 10^5 ^re-samples. Hence in this case positive data was only obtained when limiting the analysis to evolutionarily conserved segments of the promoters. The analysis of the BOYER_NANOG_OCT4_SOX2 data set, that contains genes that bind all three transcription factors, produced top ranking E- and P-scores for the OCT related bindings site whereas the NANOG site showed moderate significance when using the phylogenetic foot printing approach. On the other hand, the OCT binding sites showed top scores in the BOYER_NANOG data set (blue in Additional file 1, Figure S1A) and conversely, NANOG showed top scores in the BOYER_OCT4 data set (blue in Additional file 1, Figure S1B), indicating, as expected, a significant overlap in the binding sites for these two transcription factors. In addition to the BOYER_E2F4 data we also analyzed the XU_E2F ChIP-Chip data using a 10^6 ^resampling procedure in which E2F1, E2F1DP1, E2F4DP2, E2F1DP1EB TFBS all obtained maximum E- and P-scores, i.e. equal to 6. The SMEENK_TP53 ChIP-Chip data did not give as clear cut results as the previous ChIP-Chip data. The promoter analysis of TP53 binding genes resulted in the top ranking E-score (~3.3) but not the top ranking P-score for TP53 (Additional file 1, Figure S1C) even though the TP53 binding motif was present in 71% of the promoters (Additional file 1, Figure S1D).

We analyzed the ZELLER_MYC ChIP-Chip data in three different ways. First all genes reported to bind MYC, then the genes that bind MYC but lack the Ebox, and then the genes that bind MYC and contain an Ebox were included in the analysis. In the first analysis the MYCMAX binding site was the most significant both with respect to E- and P-scores (Additional file 2, Figure S2A). The same analysis of MYC binding genes lacking Ebox showed no significant enrichment of MYC related binding sites (Additional file 2, Figure S2B), whereas the analysis of Ebox containing genes showed significant E- and P-scores for all MYC related binding sites (Additional file 2, Figure S2C). In fact all six, NMYC, MYCMAX, MAX, MYC, MAX, and CMYC, as well EBOX, showed maximum E- and P-scores of 5. In a subsequent analysis using a 10^6 ^re-sample approach NMYC, MYCMAX, MAX, and CMYC, as well as EBOX binding sites obtained maximum E- and P-scores of 6, underscoring the enrichment of these sites in the promoters of MYC binding genes with Eboxes (Additional file 2, Figure S2D). As this analysis was limited to the -1500 to +500 regions of the promoters one may tentatively conclude that Ebox containing genes preferentially show MYC binding sites in the close vicinity of the TSSs whereas genes lacking Eboxes may bind the MYC transcription factors outside the -1500,+500 regions, or use an as yet not identified binding motif.

**Figure 2:**
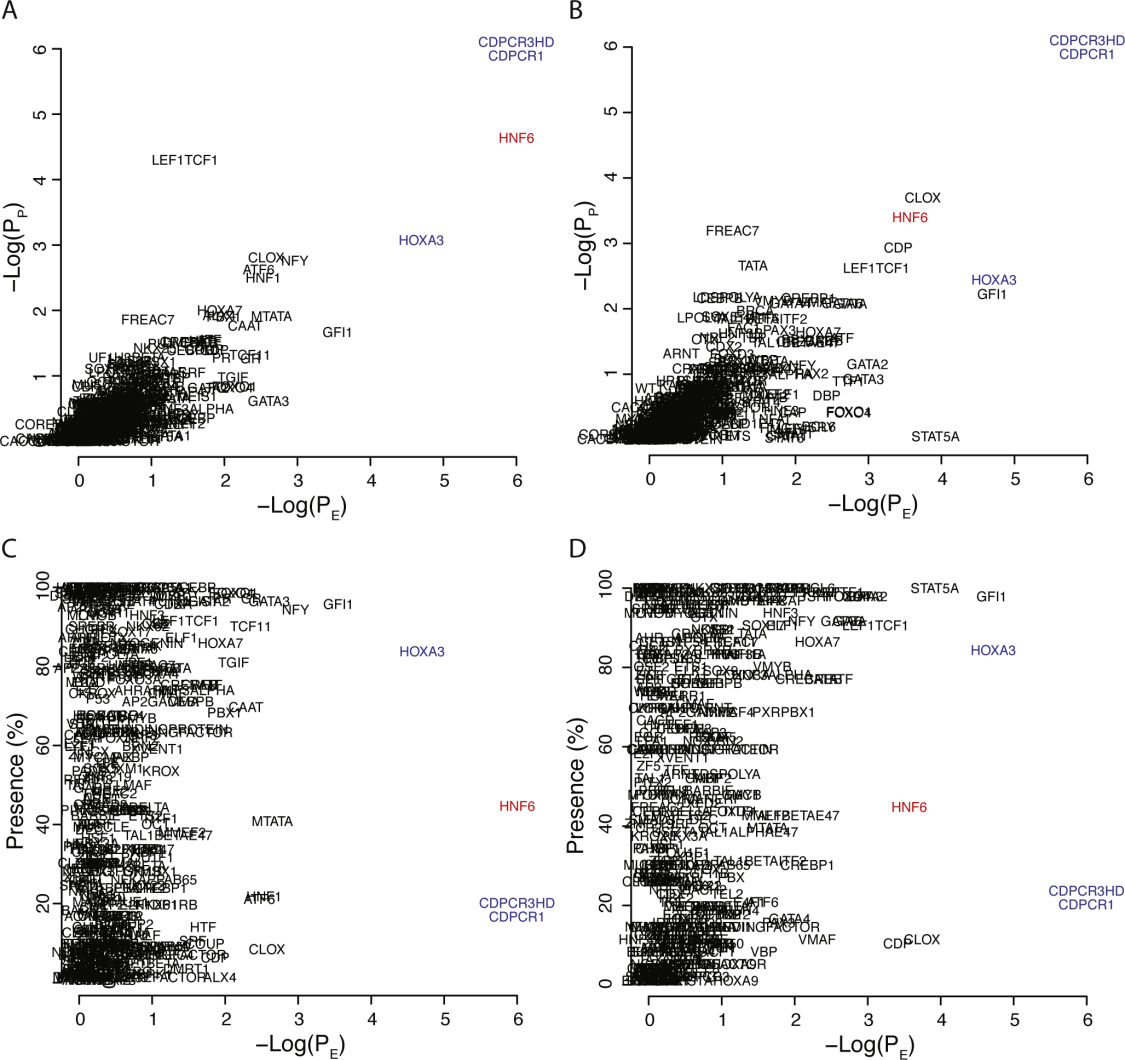
**Promoter analysis of genes binding HNF6 as determined by ChIP-Chip analysis**. Estimations of P_E _and P_P _based on 10^6 ^re-samples and the -1500,+500 portions of the promoters. A) Results for HNF6 binding genes in hepatocytes, ODOM_HNF6_Hepa B) Results for HNF6 binding genes in pancreatic cells, ODOM_HNF6_Panc. C) Results for ODOM_HNF6_Hepa plotted with E-score on the x-axis and %-presence on the Y-axis. D) Results for ODOM_HNF6_Panc plotted with E-score on the x-axis and %-presence on the Y-axis.

We then analyzed the ODOM_HNF6_Hepa and ODOM_HNF6_Panc ChIP-Chip data, Figure 2A and 2B. In both sets of genes HNF6 binding sites showed top-ranking E- and P-scores and were detected in 45% of the promoters in the Hepa and Panc gene sets respectively (Figure 2C and 2D). Interestingly, the binding sites CDPCR3HD and CDPCR1 both showed maximum E- and P-scores in both data sets. These sites were present in 18 and 21% in the HNF6-Hepa and HNF6-Panc promoters, respectively, indicating these as possible co-factors in a sub set of the genes. The data also indicated the possible role for HOXA3 in the regulation of this set of genes. HOXA3 was present in 84% of the promoters from both cell types and was among the top ranking binding sites with respect to E- and P-scores in both the Hepa and the Panc sets of HNF6 binding genes.

**Figure 3:**
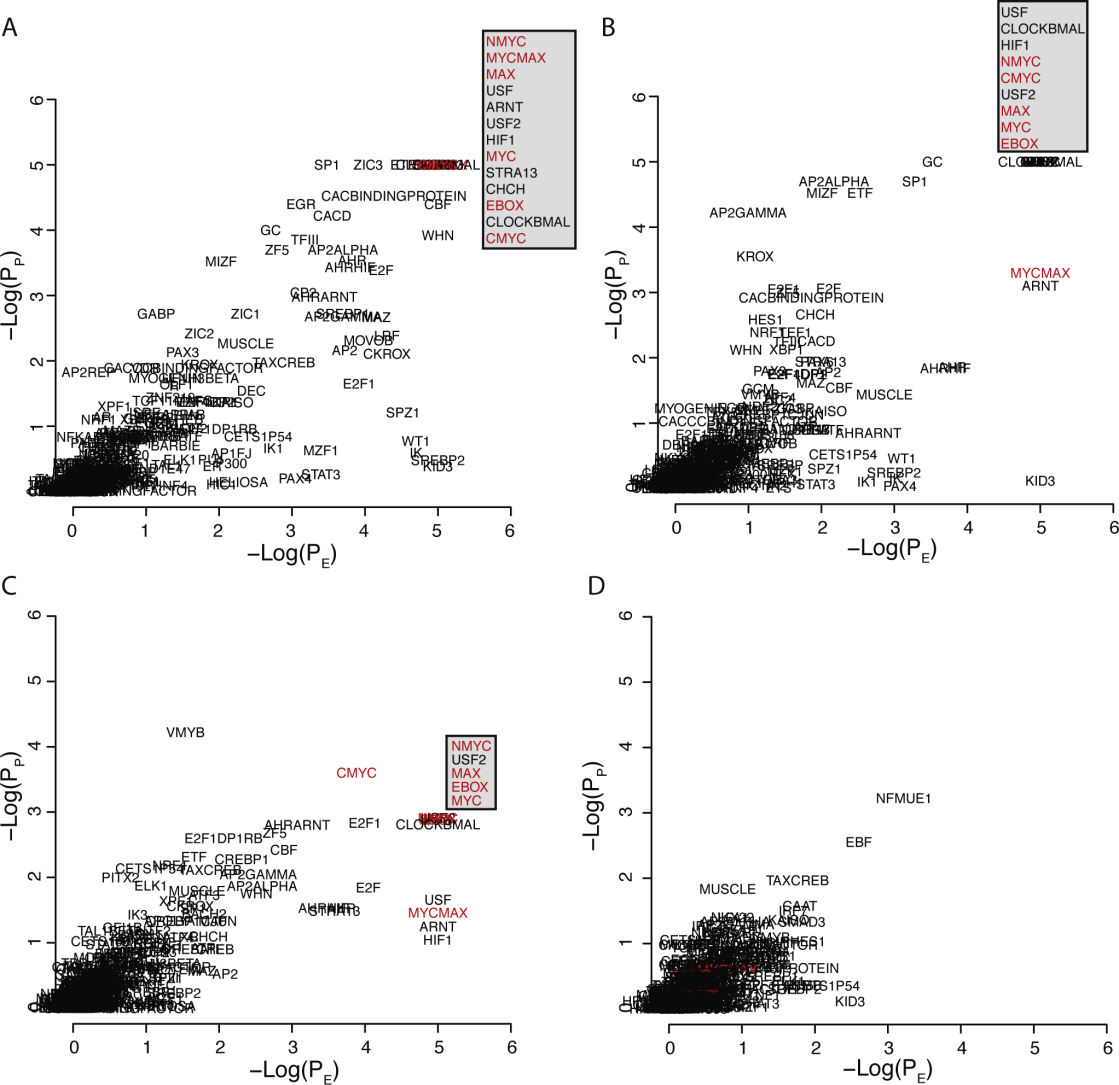
**Promoter analysis of MYC gene signatures obtained from MSigDB using the -1500,+500 portions of the promoters and 10^5 ^re-samples for estimations of P_E _and P_P_**. A) Results for the FERNANDEZ_MYC_TARGETS signature. Insert, motifs with maximum -Log(P_E_) and -Log(P_P_) values, MYC related motifs in red, B) Results for the MYC_ONCOGENE_SIGNATURE signature. Insert, motifs with maximum -Log(P_E_) and -Log(P_P_) values, MYC related motifs in red C) Results for the MYC_TARGETS signature. Insert, motifs with maximum -Log(P_E_) and -Log(P_P_) values, MYC related motifs in red d) Results for the LEE_MYC_UP signature. MYC related motifs in red. All signature designations are as in MSigDB.

**Figure 4:**
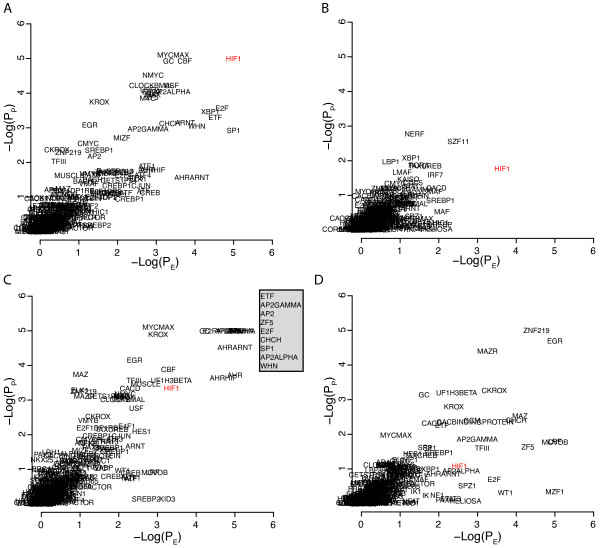
**Promoter analysis of hypoxia related gene signatures obtained from MSigDB using the -1500,+500 portions of the promoters and 10^5 ^re-samples for estimations of P_E _and P_P_**. A) Results for the MENSE_HYPOXIA_UP signature. B) Results for the HIF_TARGETS signature. C) Results for the HYPOXIA_NORMAL_UP signature. Insert, motifs with maximum -Log(P_E_) and -Log(P_P_) values. D) Results for the MANALO_HYPOXIA_UP signature. All signature designations are as in MSigDB.

**Figure 5:**
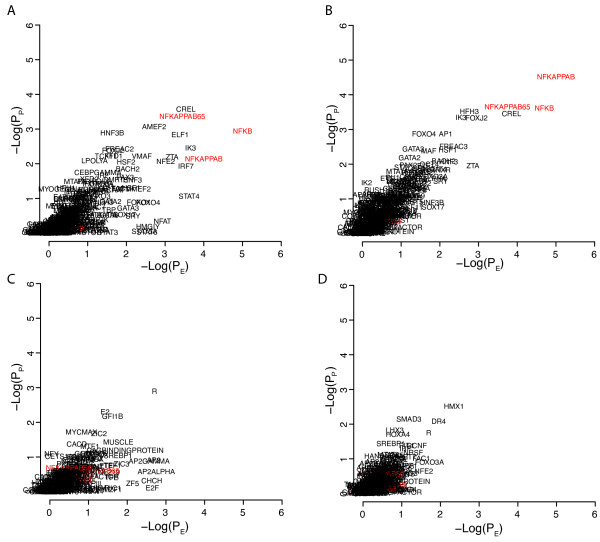
**Promoter analysis of the HINATA_NFKB_UP and HANSON_NFKAPPB_IND gene signatures using 10^5 ^re-samples for estimations of P_E _and P_P_**. Results for the HINATA_NFKB_UP signature using A) the -1500,+500 portions of the promoters and B) using phylogenic foot printing and the -5000,+1000 portions of the promoters. Results for the HANSON_NFKAPPB_IND signature using C) the -1500,+500 portions of the promoters and D) using phylogenic foot printing and the -5000,+1000 portions of the promoters. NFKB related motifs in red. All signature designations as in MSigDB.

To further explore the strategy we analyzed gene signatures obtained from the MSigDB database associated with induced expression of various transcription factors or processes (hypoxia) known to involve specific transcription factors. We downloaded four gene sets related to MYC gene expression, FERNANDEZ_MYC_TARGETS, MYC_ONCOGENE_SIGNATURE, MYC_TARGETS, and LEE_MYC_UP. The genes in the respective gene sets were analyzed in the -1500,+500 portions of the promoters using a 10^5 ^resampling procedure (Figure 3). All MYC related binding sites, MYCMAX, MYC, NMYC, CMYC, MAX, and EBOX, showed maximum E- and P-scores (equal to 5) in the FERNANDEZ_MYC_TARGETS and all but MYCMAX in the MYC_ONCOGENE_SIGNATURE gene set. All but CMYC showed maximal E scores in the MYC_TARGETS gene set whereas the P scores were moderate but still among the top ranking. None of the MYC related binding sites showed high scores in the LEE_MYC_UP gene set, in fact all of them showed E- and P-scores below 1. A possible explanation for the negative results obtained for the LEE_MYC_UP signature could be that this signature is dominated by late/secondary MYC targets not directly regulated by the MYC protein. An indication that this may be the case was the finding that only 27% of the promoters in the LEE_MYC_UP gene set contained EBOX motifs whereas these were seen in 70% of the promoters in the FERNANDEZ_MYC_TARGETS gene set. Essentially the same results were obtained when repeating the analysis using the phylogenic foot printing approach, including the negative results for the LEE_MYC_UP gene set (Additional file 3, Figure S3). We performed similar analyses of four hypoxia related gene signatures (Figure 4). The HIF1 binding site was the single site that showed maximum E/P scores in the MENSE_HYPOXIA_UP gene set. The HIF1 site showed the top ranking E-score and was among the top ranking with respect to the P-score in the HIF1_TARGETS gene set, showed moderate E- and P-scores (3 to 4) in the HYPOXIA_NORMAL_UP gene set, and showed low scores (<3) in the MANALO_HYPOXIA_UP gene set. Hence, the analysis indicated HIF1 as the most important binding site in two of the gene sets. Repeating the analysis using the phylogenic foot printing approach further emphasized the importance of HIF1 sites, particularly in the HYPOXIA_NORMAL_UP gene set, but did not improve the results for the MANALO_HYPOXIA_UP set of genes (Additional file 4, Figure S4). As for the LEE_MYC_UP signature, the poor results for the MANALO_HYPOXIA_UP gene set could possibly be explained by the presence of a hypoxia late response signature. We also noticed that whereas the HIF1 site was one of the top ranking TFBS in all the MYC signatures investigated that also showed MYC targets as top-ranking, MYC TFBSs were not top-ranking in the hypoxia related signatures. The HINATA_NFKB_UP gene set showed top ranking E- and P-scores for the NFKB, NFKAPPB, and NFKAPPB65 binding sites using the -1500,+500 regions (Figure 5A). The NFKAPPB50 motif was however not significant. The importance of the NFKB related TFBS for this set of genes was further emphasized using the phylogenic foot printing approach (Figure 5B). Poor results were however obtained for the HANSON_NFKAPPB_IND gene set using both approaches (Figure 5C and 5D). The analysis of the KANNAN_TP53_UP gene signature indicated P53 as the most significantly enriched binding site with an E-score close to 4. Even though the P-score was non-convincing (~1), the TP53 site was present in close to 80% of the promoters. This type of results i.e., moderate E-Scores and low P-scores but high %-presence, was also obtained for the SMEENK_TP53 ChIP-Chip data and indicates that TP53 binding sites are frequently seen in promoters albeit at low numbers for each promoter. Additional MSigDB signatures that performed well were E2F3_ONCOGENETIC_SIGNATURE generating E- and P-scores of 5 for E2F1, E2F, and E2F1DP1RB site, respectively; HALMOS_CEBP indicating CEBP and CEBPGAMMA as the two top two binding sites with respect to E- and P-scores when applying phylogenic foot printing, and the BILD_MYC signature, that included EBOX, MYC, MAX, CMYC, and MYCMAX among the top ten ranking together with USF, USF2, HIF1, ARNT, and CLOCKBMAL, and BILD_E2F3, that included E2F1, E2F, E2F1DP1, and E2F1DP1RB among the 15 binding sites with E- and P-scores equal to 5.

### Identification of sets of co-occurring TFBS and defining TFBS based gene signatures

**Figure 6:**
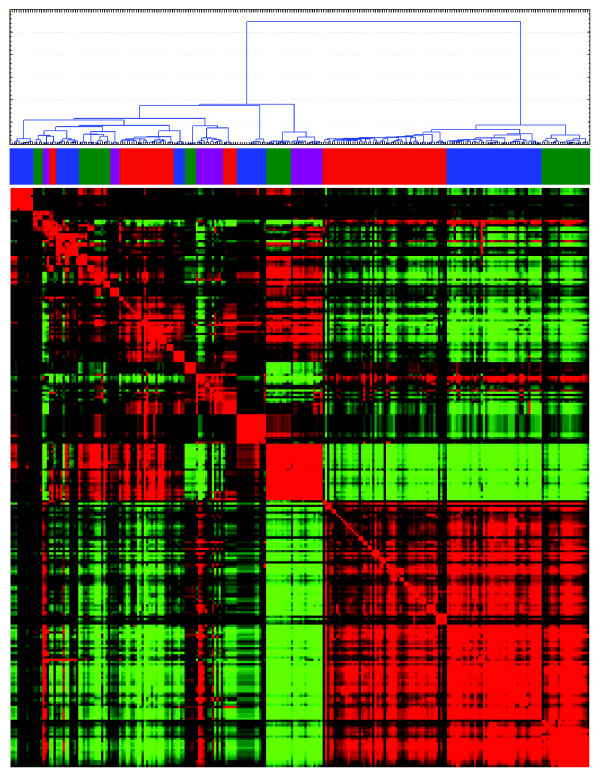
**Identification of correlated TFBS as identified using the -1500,+500 portions of the promoters**. A hierarchical cluster analysis with adjoined heat map of correlations of the portion of the TFBS that showed sizeable correlations and indicated in Additional file 5, Figure S5.

**Figure 7:**
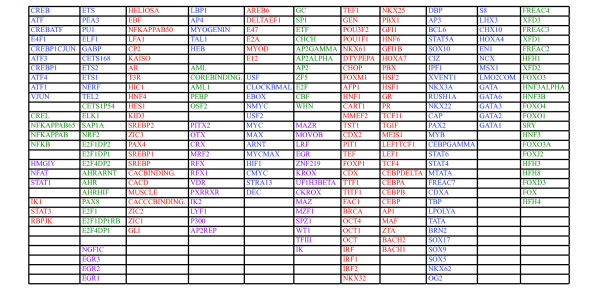
**Identified clusters of TFBS using the -1500,+500 portions of the promoters**. Color codes listed in the order left to right as in the dendrogram in Figure 6.

It is well established that most transcription factors do not act in solitude but in a cooperative fashion with other factors. Hence, co-regulated genes are expected to share more than one TFBS in their promoters, as was, e.g., seen in the above analyses of the MSigDB gene sets. To gain new knowledge on such possible combinations we used the -1500 to +500 TFBS/promoter database to produce a correlation matrix among the TFBS's using the number of instances for each TFBS in each promoter as variables. This matrix was subsequently re-ordered using hierarchical clustering analysis (HCA; Additional file 5, Figure S5). The obtained re-ordered correlation matrix did indeed show distinct features. Apart from TFBS's showing strong and significant correlations, a portion of the TFBS showed no or very low correlations. In Figure 6 the analysis has been limited to subset of TFBS that showed the most varying correlations (indicated in Additional file 5, Figure S5). Based on the hierarchical clustering several tentative groups of co-occurring TFBS could be defined (Figure 6 and 7). Interestingly, strong negative correlations were also detected in the data, particularly between the TEF1_BACH1, DBP_GATA, and FREAC_HFH clusters and the GC_WHN and MAZR_IK TFBS clusters suggesting a selection against the co-occurrence of these groups of TFBS in gene promoters. No negative correlations were however detected when the analysis was repeated using the evolutionarily conserved portions of the -1500,+500 promoter regions (data not shown).

**Figure 8:**
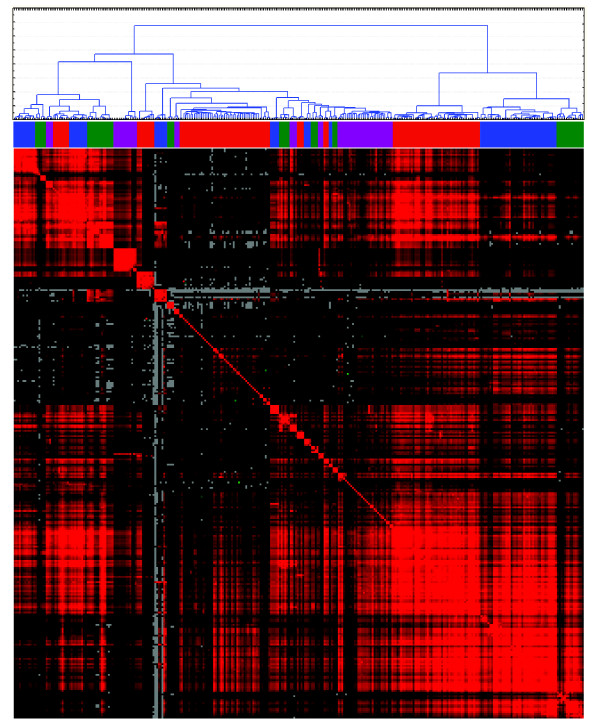
**Identification of correlated TFBS as identified using the -5000,+1000 portions of the promoters and phylogenic foot printing**. A hierarchical cluster analysis with adjoined heat map of correlations of the portion of the TFBS that showed sizeable correlations and indicated in Additional file 7, Figure S6.

**Figure 9:**
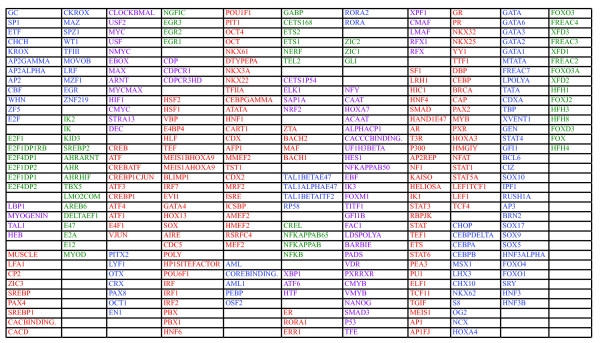
**Identified clusters of TFBS using the -5000,+1000 portions of the promoters and phylogenic foot printing**. Color codes listed in the order left to right as in the dendrogram in Figure 8.

We then produced gene signatures based on groups of correlated TFBS as defined by the HCA. To evaluate the coherence of the top ranking genes in the respective gene lists the top 2000 genes were analyzed for GO category enrichment. Of the 18 TFBS clusters listed in Figure 8, twelve showed significant enrichment (Bonferroni corrected p < 0.05) of gene ontology terms (Table 2 and Additional file 6, Table S1). Hence, most of the TFBS based gene signatures were significant for specific biological processes, including as diverse processes as regulation of transcription, intracellular signaling cascade, and immune response. The above analysis was repeated with the TFBS/promoter database based on phylogenic foot printing and the -5000,+1000 portions of the promoters. Again several clusters of co-occurring TFBS was identified and the most varying TFBS were selected for further analysis (indicated in Additional file 7, Figure S6). The subsequent HCA identified 26 TFBS clusters (Figure 8 and 9) of which 20 showed significant enrichment of specific GO categories (Additional file 8, Table S2). These included top ranking GO categories as diverse as regulation of transcription, cell adhesion, and neurogenesis.

**Table 2:** GO category analysis of TFBS cluster gene signatures.

Signature	Gene Category	EASE score
CREB_VJUN	nucleobase\, nucleoside\, nucleotide and nucleic acid metabolism	6.03E-12
CREL_NFKB	immune response	2.06E-07
HMGIY_STAT	Ns	ns
IK1_RBPJK	nucleobase\, nucleoside\, nucleotide and nucleic acid metabolism	8.57E-06
ETS_TEL2	Ns	ns
CETS_E2F4	nucleobase\, nucleoside\, nucleotide and nucleic acid metabolism	4.13E-20
NGFC_EGR	Ns	ns
HELIOSA_GLI	intracellular signaling cascade	1.21E-06
LBP1_HEB	Ns	ns
AML	response to biotic stimulus	3.85E-10
PITX2_AP2	protein metabolism	3.07E-05
AREB6_E12	Ns	ns
MYC	Ns	ns
GC_WHN	regulation of transcription	3.67E-16
MAZR_IK	regulation of transcription\, DNA-dependent	7.14E-29
TEF1_BACH	response to external stimulus	4.19E-16
DBP_GATA	response to external stimulus	1.14E-18
FREAC_HFH	response to external stimulus	5.39E-07

There are arguments for using relatively short intervals as the definition of a promoter as well as for longer intervals but then limit the analysis to evolutionarily conserved regions. Both approaches have advantages and limitations. The above analyses of ChIP-Chip and MSigDB gene signatures, as well as that of co-occurring TFBS, show that similar but not always identical results may be obtained using either approach. To capitalize on the benefits of both approaches we produced a third TFBS/promoter database that takes into consideration the -1500,+500 portions of the promoters as well as the -5000 to -1500 and the +500 to +1000 evolutionarily conserved portions. This produced a TFBS/promoter database consisting of 13 096 genes. We used this database to identify co-occurring TFBS using the QT-clust algorithm, a more stringent method than manual inspection of HCA dendrograms. This algorithm produced 58 groups of at least two co-occurring TFBS (Table 3 and Additional file 9, Table S3). Many of these were identical, overlapped, or were parts of clusters also identified by the less stringent HCA approach. As above, ranked gene list based on the normalized number of TFBS hits in each gene promoter were produced and the top 1300 genes in each TFBS gene signature were subjected to analysis using the Ingenuity software. In Table 4 results obtained for the Ingenuity categories "Molecular and Cellular Functions", "Physiological System Development and Function" and "Top Canonical Pathways" are listed for 10 of the most biologically coherent TFBS clusters. Intriguingly, of the 10 TFBS clusters in Table 4, three were involved in "Nervous System Development" or "Function and Axonal Guidance Signaling" and five in immune response, which may indicate that these processes are orchestrated by a several combinations of transcription factors. Taken together, both the HCA and the QT-clust formed clusters of co-occurring TFBS that defined gene signatures with distinct biological properties.

**Table 3:** The twelve largest clusters of TFBS formed after QT-clust analysis

QTCluster_1	QTCluster_2	QTCluster_3	QTCluster_4	QTCluster_5	QTCluster_6
NKX62	USF	GC	CKROX	CREB	LMO2COM
OG2	CLOCKBMAL	ETF	SPZ1	ATF	AREB6
FOXO4	HIF1	AP2GAMMA	WT1	CREBP1CJUN	E47
FOXO1	NMYC	MOVOB	MZF1	ATF4	E2A
BCL6	ARNT	AP2	MAZ	ATF3	MYOD
SOX10	USF2	LRF	TFIII	CREBP1	E12
STAT5A	MYCMAX	CHCH	IK		
S8	MAX	SP1			
SRY	MYC	AP2ALPHA			
PAX2	EBOX				
STAT4					
CDXA					
TBP					
					
**QTCluster_7**	**QTCluster_8**	**QTCluster_9**	**QTCluster_10**	**QTCluster_11**	**QTCluster_12**
HNF3ALPHA	GATA	LBP1	AML	HELIOSA	CETS1P54
FOXJ2	GATA6	AP4	COREBINDINGFACTOR	ETS	SAP1A
FOXO3A	GATA3	TAL1	PEBP	IK1	ELK1
HNF3B	GATA2	MYOGENIN	OSF2	STAT3	NRF2
HFH3	GATA1	HEB	AML1		
HNF3	RUSH1A				

**Table 4:** The ten most coherent TFBS-cluster gene signatures according to the Ingenuity software^a^

	p-value
**GC, ETF, AP2GAMMA, MOVB, AP2, LRF, CHCH, SP1, AP2LAPHA**	
Cellular Development (MCF)	4.32E-13
Organ Development (PSDF)	2.43E-14
Wnt/b-catenin Signaling(CP)	3.90E-10
	
**CKROX, SPZ1, WT1, MZF1, MAZ, TFIII, IK**	
Cellular Development (MCF)	3.97E-12
Tissue Development (PSDF)	1.23E-12
Wnt/b-catenin Signaling (CF)	1.51E-05
	
**AML, COREBINDINGFACTOR, PEBP, OSF2, AML1**	
Cell-To-Cell Signaling and Interaction (MCF)	5.29E-05
Hematological System Development and Function (PSDF)	3.08E-05
Immune Cell Trafficking (PSDF)	3.08E-05
	
**CACBINDINGPROTEIN, CACD, PAX4, SREBP1**	
Cellular Development (MCF)	6.48E-12
Organismal Development (PSDF)	4.92E-16
Axonal Guidance Signaling (CP)	4.26E-05
	
**HMGIY, STAT6, STAT1, NFAT**	
Cellular Development (MCF)	2.43E-08
Nervous System Development and Function (PSDF)	6.45E-18
Axonal Guidance Signaling (CF)	4.24E-04
	
**CEBPDELTA, CEBPB, CEBPA**	
Antigen Presentation (MCF)	4.28E-04
Humoral Immune Response (PSDF)	2.98E-04
T Helper Cell Differentiation (CP)	1.61E-03
	
**ZF5, E2F, KROX**	
Gene Expression (MCF)	6.32E-22
Organ Development (PSDF)	5.59E-16
Axonal Guidance Signaling (CP)	1.13E-12
	
**ZTA, BACH2, BACH1**	
Antigen Presentation (MCF)	1.35E-06
Cell-mediated Immune Response (PSDF)	1.35E-06
NF-kB Signaling (CP)	1.71E-03
	
**CREL, NFKAPPAB65**	
Cell-To-Cell Signaling and Interaction (MCF)	2.73E-05
Cell-mediated Immune Response (PSDF)	1.81E-06
NF-kB Signaling (CP)	4.28E-06
	
**MAZR, ZNF219**	
Cellular Development (MCF)	9.61E-13
Nervous System Development and Function (PSDF)	1.23E-10
Axonal Guidance Signaling (CP)	1.40E-07

^a ^Only the top categories with respect to p-value are given

## Discussion

The backbone of the present investigation is the construction of the TFBS/promoter databases that we used for two purposes; as a background files when analyzing promoters for significant TFBS and as possible sources for investigating relationships among TFBS. As the definition of a promoter may vary from type of investigation and scientific questions asked, an advantage of the presented tool is that variant TFBS/promoter databases may be produced readily. In the present investigation we used three types of databases each based on different promoter definitions; regions derived from short segments upstream of the TSS, conserved regions derived from large upstream segments, and a combination of these two. We used two measures for significance, significantly enriched (E) and significantly present (P) TFBS, and rank statistics based on a resampling procedure to estimate the corresponding p-values. The E and P measures capture two aspects of TFBS significance, the recognition that genes regulated by a specific transcription factor tend to have several recognition sites for that particular factor and that genes regulated by a specific transcription factor are expected to have at least one binding site each for that factor. The advantage of estimating p-values by a re-sampling method is that no assumptions regarding the underlying distribution of TFBS or of promoter DNA sequences has to be made. Even though we used a simple rule to score promoters (genes), *i.e*., the number of high scoring motifs per promoter, biologically relevant and intriguing results were obtained. Furthermore, as we use gene sets as the unit for investigation, and not individual promoters, the inclusion of false positive sites for individual genes identified with PWMs will have limited effect on the final results. We showed that the presented strategy could faithfully reproduce several ChIP-Chip results, and hence validate the strategy. Nonetheless, there were some ChIP-Chip data that the strategy could not reproduce, *e.g*., Palomero et al. [9] (TAL1 targets) and Reed et al. [10] (SREBP1 targets). There might be more than one explanation for these discrepancies, *e.g*., ChIP-Chip data most likely contain false positives to a varying degree and the PWM database employed, TRANSFAC, may not be complete. An indication that the latter may be the case is the fact that several ChIP-Chip and ChIP/PET investigations have led to the discovery of new consensus binding sites *e.g*., both Smeenk et al. [11] and Wei et al. [12] present new TP53 motifs based on whole genome ChIP analyses. It is thus likely that future ChIP-Chip and ChIP/PET analyses will describe additional motifs for other transcription factors as well. Discrepant results for MSigDB derived gene signatures may be caused by several factors. Apart from the fact that not all possible PWMs may be present in the TRANSFAC database, an additional complication is that the response to a given perturbation may vary with the experimental procedures used, *e.g*., the gene signature response may vary with time and thus include secondary targets to varying degrees. An additional factor that may reduce the E- and P-scores is that most signatures in MSigDB have been obtained by micro array experiments in which not all possible genes were included in the analysis. Consequently, estimations by rank statistics using the whole genome as a reference, as was performed, may underestimate and skew the actual E- and P-scores. Moreover, in some instances it may be beneficial to use a reference matched to some characteristics of the gene signature, e.g., GC-content [13]. Nonetheless, several of the gene signatures from the MSigDB produced consistent results, again showing the strength of the proposed strategy.

Apart from using the TFBS/promoter databases as references when performing promoter analyses the databases were also used as a source for investigating TFBS co-occurrences. Using hierarchical cluster analysis we were able to detect several clusters of TFBS. Similar, and often identical, clusters of TFBS were obtained when applying the phylogenic foot printing approach as well as when we used the hybrid TFBS/promoter database and an alternative clustering principle. This indicates that the observed TFBS clusters are to a certain level robust and not dependent on the algorithms used to detect them. Some of the obtained TFBS clusters were most likely caused by the presence of overlapping binding motifs, *e.g*., the AML and MYC TFBS clusters, whereas others may represent true co-occurrence in the sense that the DNA sequence of the binding motifs are unrelated. As no criterion for proximity, *i.e*., minimum distance between TFBS, was used many of the identified TFBS patterns are less constrained than so called TFBS modules, typically 100-300 bp in size. The obtained results thus suggest that long distance but within promoter combinatorics may operate among transcription factors. Interestingly, some clusters of TFBS showed negative correlations. The negative correlations were however lost when the analyses were limited to the evolutionarily conserved parts of the promoters. This makes sense if selection against co-occurrence results in disruption of the respective TFBS as this ultimately would result in a degenerated sequence and no sequence conservation. Such selection could possibly act to minimize contradicting regulatory signals caused by, *e.g*., spurious expression of transcription factor genes, or to keep different cellular states/processes well separated from a regulatory point of view. The target genes for the respective TFBS clusters represented in almost all cases biologically coherent sets of genes, as determined by GO term enrichment analyses or the Ingenuity software, and represented highly divergent cellular processes. Hence, we conclude that the identified TFBS clusters, and their associated gene signatures, are of biological relevance.

We are aware of several limitations of the above analyses. For instance, the most appropriate way to identify TFBS that co-regulate genes might not be to investigate global correlation as this measure includes mutual absence in the comparison. An alternative measure of similarity could have been the Jaccard algorithm [14]. We also applied the same thresholds for all clusters when selecting genes associated with the specific TFBS clusters, the top 2000 and 1300 respectively. As the number of promoters positive for a given TFBS varies with the particular TFBS, an alternative selection criterion would have been to select a given fraction of the positive promoters (genes), *e.g*., the top 80% of the positive genes, as associated with the signature, which would have produced target gene lists of varying sizes; an option that could easily be biologically motivated. As an alternative to the sum of the normalized hits used in the preset investigation various Boolean conditions could also have been employed or added as a limiting criteria. Nevertheless, most of the identified TFBS gene signatures were associated with coherent cellular processes and made biological sense. To facilitate future investigations we will provide the software used in this available at http://cbbp.thep.lu.se/~markus/software/SMART/.

## Conclusions

We have provided a flexible tool to produce TBFS/promoter databases. We have described a approach to identify significant TFBS in gene sets by use of a resampling strategy and the introduction of a new measure of significance, significantly present (P_P_). We apply this approach to several published ChIP-Chip data and curated gene signatures obtained from MSigDB and show that the strategy produces consistent results and hence may be useful in the analysis of gene expression profiles or ChIP-Chip data. In addition, we have shown that TFBS form stable clusters of co-occurring TFBS that in a consorted fashion seems to regulate sets of genes associated with highly specific cellular processes.

## Methods

### Data sets

We selected published ChIP-Chip data sets for validation using the criteria that the data should be easy to access and that approximate positions of the binding sites relative the TSS of the genes were available. For E2F4, NANOG, and OCT4 ChIP-Chip target genes we used the data provided by Boyer et al. [15], named BOYER_E2F4, BOYER_NANOG, and BOYER_OCT4 in the present investigation. We also included a list of genes that by ChIP-Chip analysis were shown to simultaneously bind NANOG, OCT4, and SOX2 provided by Boyer at al., BOYER_NANOG_OCT4_SOX2. The analyses were limited to genes that contained targets within 8 kb upstream of the TSS. ChIP-Chip data from Xu et al. [16] were used to produce a list of genes (XU_E2F) with strong E2F binding. For MYC ChIP-Chip target genes we used data provided by Zeller et al. [17] but only included genes with 5' locations of ChIP-Chip targets (ZELLER_MYC). Only genes with reported ChIP-Chip targets within the -1500 to TSS region were included. For analyses of HNF6 target genes in hepatocytic and pancreatic cells the data provided by Odom et al. [18] was used (ODOM_HNF6_Hepa and ODOM_HNF6_Panc). TP53 ChIP-Chip target genes were obtained from the Smeenk et al. [11] ChIP-Chip data (SMEENK_TP53). Two SMEENK_TP53 data sets were produced, one including genes with ChIP-Chip targets within the -1500 to +500 promoter segment and one including genes with targets within the -5000 to +1000 promoter segment. Curated gene sets for various conditions were obtained from the MSigDB database [19]. Gene set designations are the same as in the MSigDB database. Gene signatures for MYC and E2F3 were obtained from Bild et al. [20], BILD_MYC and BILD_E2F3, respectively.

### Clustering algorithms

We used hierarchical clustering with 1-Pearson as a distance measure and average-linkage for agglomeration in the initial analyses. Groups of TFBS that behaved similarly were identified by manual inspection. We also used the more stringent QT Clust (Quality Cluster algorithm) modified from Heyer et al. [21]. QT Clust proceeds by forming a candidate cluster with the first TFBS as a seed and grouping TFBS's with the highest correlation iteratively in a way that minimizes the cluster diameter d, until no further genes may be added without exceeding a predetermined d-value. This procedure is performed with all TFBS in the data set as a seed. The largest cluster is then retrieved and the procedure repeated with the remaining TFBS on the list until no further clusters may be formed. This makes sure that the largest and most coherent clusters of TFBS are formed. We used a threshold diameter of 0.5 based on Pearson correlation and a minimal cluster size of 2 members. Identified TFBS cluster gene signatures were evaluated either with the EASE [22] or the Ingenuity software [23]. To produce gene signatures for groups of correlated TFBS we first normalized the number of hits per promoter by dividing with the total number of detected hits for each TFBS. Then the sum of the normalized hits for gene members in a given TFBS cluster was calculated for each gene promoter, and finally the genes were ranked according the sum of normalized hits. This produced gene lists composed of 18 377 genes using the TFBS/promoter database based on the -1500 to +500 genomic intervals and 13 177 when using the phylogenetic foot printing approach, in which the top ranking genes will have the largest number of normalized hits for binding sites in their promoters.

## Authors' contributions

SV wrote the software and produced all the background files, performed some of the bioinformatical analyses, and wrote parts of the manuscript; MR explored the software beta-version, suggested several improvements, and applied it to additional data sets; MH, conceived the investigation, performed most of the bioinformatical analyses, and wrote the major part of the manuscript. All authors read and approved the final manuscript.

## Supplementary Material

Additional file 1 **Figure S1. Promoter analysis of genes binding NANOG, OCT4, and TP53 as determined by ChIP-Chip analyses**. All results based on 10^5 ^re-samples for estimations of P_E _and P_P _a) analysis of promoters of NANOG binding genes using phylogenic foot printing of the -5000,+1000 portions of the promoters. b) Analysis of promoters of OCT4 binding genes using phylogenic foot printing of the -5000,+1000 portions of the promoters. All TBFS names having PE values equal to 5 except NANOG, OCT, OCT1, and OCT4 omitted from the graph for clarity. c) Analysis of promoters of TP53 binding genes using the -1500,+500 portions of the promoters. d) Analysis of promoters of TP53 binding genes using phylogenic foot printing of the -5000,+1000 portions of the promoters. Click here for file

Additional file 2 **Figure S2. Promoter analysis of genes binding MYC as determined by ChIP-Chip analysis**. a) Analysis of promoters of all MYC binding genes in the ZELLER_MYC data. b) Analysis of promoters of MYC binding genes lacking EBOXes c) Analysis of promoters of MYC binding genes with EBOXes. d) Analysis of promoters of MYC binding genes with EBOXes using 10^6 ^instead of 10^5 ^re-samples to compute P_E _and P_P_. Click here for file

Additional file 3 **Figure S3. Promoter analysis of MYC gene signatures obtained from MSigDB, using the -5000,+1000 portions of the promoters, phylogenic foot printing, and 10^5 ^re-samples for estimations of P_E _and P_P_**. a) Results for the FERNANDEZ_MYC_TARGETS signature. b) Results for the MYC_ONCOGENE_SIGNATURE signature. c) Results for the MYC_TARGETS signature. d) Results for the LEE_MYC_UP signature. All signature designations are as in MSigDB. Click here for file

Additional file 4 **Figure S4. Promoter analysis of hypoxia related gene signatures obtained from MSigDB, using phylogenic foot printing and 10^5 ^re-samples for estimations of P_E _and P_P_**. a) Results for the MENSE_HYPOXIA_UP signature. b) Results for the HIF_TARGETS signature. c) Results for the HYPOXIA_NORMAL_UP signature. d) Results for the MANALO_HYPOXIA_UP signature. All signature designations are as in MSigDB. Click here for file

Additional file 5 **Figure S5. Analysis of correlations among TFBS obtained using the -1500,+500 portions of the promoters**. Heat map obtained after reorganization with hierarchical cluster analysis. The portion of TFBS used for further analyses in box. Click here for file

Additional file 6 **Table S1. GO category analysis of TFBS cluster gene signatures obtained after hierarchical cluster analysis using the -1500,+500 portions of the promoters**. Bonferroni corrected EASE scores < 0.05 considered significant. Click here for file

Additional file 7 **Figure S6. Analysis of correlations among TFBS obtained using the -5000,+1000 portions of the promoters and phylogenic foot printing**. Heat map obtained after reorganization with hierarchical cluster analysis. The portion of TFBS used for further analyses in box. Click here for file

Additional file 8 **Table S2. GO catagory analysis of TFBS cluster gene signatures obtained after hierarchical cluster analysis using the -5000,+1000 portions of the promoters and phylogenic foot printing**. Bonferroni corrected EASE scores < 0.05 considered significant. Click here for file

Additional file 9 **Table S3. List of all 58 TFBS clusters formed after QT-clust analysis**. Click here for file

## References

[B1] Kel AE Gössling E Reuter I Cheremushkin E Kel-Margoulis OV Wingender E MATCH: A tool for searching transcription factor binding sites in DNA sequences Nucleic Acids Res 2003 31 13 3576 9 10.1093/nar/gkg585 12824369 PMC169193

[B2] Cartharius K Frech K Grote K Klocke B Haltmeier M Klingenhoff A Frisch M Bayerlein M Werner T MatInspector and beyond: promoter analysis based on transcription factor binding sites Biochem Biophys Res Commun 2005 334 2 516 23 10.1016/j.bbrc.2005.06.120 15860560

[B3] Schug J Using TESS to predict transcription factor binding sites in DNA sequence Curr Protoc Bioinformatics 2008 Chapter 2 Unit 26 18428685 10.1002/0471250953.bi0206s21

[B4] Bryne JC Valen E Tang MH Marstrand T Winther O da Piedade I Krogh A Lenhard B Sandelin A JASPAR, the open access database of transcription factor-binding profiles: new content and tools in the 2008 update Nucleic Acids Res 2008 36 Database D102 6 Epub 2007 Nov 15 18006571 10.1093/nar/gkm955 PMC2238834

[B5] Wingender E Dietze P Karas H Knüppel R TRANSFAC: a database on transcription factors and their DNA binding sites Nucleic Acids Res 1996 24 1 238 41 10.1093/nar/24.1.238 8594589 PMC145586

[B6] Aerts S Thijs G Coessens B Staes M Moreau Y De Moor B TOUCAN: Deciphering the Cis-Regulatory Logic of Coregulated Genes Nucl Acids Res 2003 31 1753 1764 10.1093/nar/gkg268 12626717 PMC152870

[B7] Karanam S Moreno CS CONFAC: automated application of comparative genomic promoter analysis to DNA microarray datasets Nucleic Acids Res 2004 32 W475 84 10.1093/nar/gkh353 15215433 PMC441491

[B8] Kim SY Kim Y Genome-wide prediction of transcriptional regulatory elements of human promoters using gene expression and promoter analysis data BMC Bioinformatics 2006 7 330 10.1186/1471-2105-7-330 16817975 PMC1586028

[B9] Palomero T Odom DT O'Neil J Ferrando AA Margolin A Neuberg DS Winter SS Larson RS Li W Liu XS Young RA Look AT Transcriptional regulatory networks downstream of TAL1/SCL in T-cell acute lymphoblastic leukemia Blood 2006 108 3 986 92 Epub 2006 Apr 18 10.1182/blood-2005-08-3482 16621969 PMC1895859

[B10] Reed BD Charos AE Szekely AM Weissman SM Snyder M Genome-wide occupancy of SREBP1 and its partners NFY and SP1 reveals novel functional roles and combinatorial regulation of distinct classes of genes PLoS Genet 2008 4 7 e1000133 10.1371/journal.pgen.1000133 18654640 PMC2478640

[B11] Smeenk L van Heeringen SJ Koeppel M van Driel MA Bartels SJ Akkers RC Denissov S Stunnenberg HG Lohrum M Characterization of genome-wide p53-binding sites upon stress response Nucleic Acids Res 2008 36 11 3639 54 Epub 2008 May 12 10.1093/nar/gkn232 18474530 PMC2441782

[B12] Wei CL Wu Q Vega VB Chiu KP Ng P Zhang T Shahab A Yong HC Fu Y Weng Z Liu J Zhao XD Chew JL Lee YL Kuznetsov VA Sung WK Miller LD Lim B Liu ET Yu Q Ng HH Ruan Y A global map of p53 transcription-factor binding sites in the human genome Cell 2006 124 1 207 19 10.1016/j.cell.2005.10.043 16413492

[B13] Bozek K Relógio A Kielbasa SM Heine M Dame C Kramer A Herzel H Regulation of clock-controlled genes in mammals PloS One 2009 4 e4882 10.1371/journal.pone.0004882 19287494 PMC2654074

[B14] Veerla S Höglund M Analysis of promoter regions of co-expressed genes identified by micro array analysis BMC Bioinformatics 2006 7 384 10.1186/1471-2105-7-384 16916454 PMC1560170

[B15] Boyer LA Lee TI Cole MF Johnstone SE Levine SS Zucker JP Guenther MG Kumar RM Murray HL Jenner RG Gifford DK Melton DA Jaenisch R Young RA Core transcriptional regulatory circuitry in human embryonic stem cells Cell 2005 122 6 947 56 10.1016/j.cell.2005.08.020 16153702 PMC3006442

[B16] Xu X Bieda M Jin VX Rabinovich A Oberley MJ Green R Farnham PJ A comprehensive ChIP-chip analysis of E2F1, E2F4, and E2F6 in normal and tumor cells reveals interchangeable roles of E2F family members Genome Res 2007 17 11 1550 61 Epub 2007 Oct 1 10.1101/gr.6783507 17908821 PMC2045138

[B17] Zeller KI Zhao X Lee CW Chiu KP Yao F Yustein JT Ooi HS Orlov YL Shahab A Yong HC Fu Y Weng Z Kuznetsov VA Sung WK Ruan Y Dang CV Wei CL Global mapping of c-Myc binding sites and target gene networks in human B cells Proc Natl Acad Sci USA 2006 103 47 17834 9 Epub 2006 Nov 8 10.1073/pnas.0604129103 17093053 PMC1635161

[B18] Odom DT Zizlsperger N Gordon DB Bell GW Rinaldi NJ Murray HL Volkert TL Schreiber J Rolfe PA Gifford DK Fraenkel E Bell GI Young RA Control of pancreas and liver gene expression by HNF transcription factors Science 2004 303 5662 1378 81 10.1126/science.1089769 14988562 PMC3012624

[B19] Molecular Signature Database http://www.broad.mit.edu/gsea/msigdb/index.jsp

[B20] Bild AH Yao G Chang JT Wang Q Potti A Chasse D Joshi MB Harpole D Lancaster JM Berchuck A Olson JA Jr Marks JR Dressman HK West M Nevins JR Oncogenic pathway signatures in human cancers as a guide to targeted therapies Nature 2006 439 7074 353 7 Epub 2005 Nov 6 10.1038/nature04296 16273092

[B21] Heyer LJ Kruglyak S Yooseph S Exploring expression data: identification and analysis of coexpressed genes Genome Res 1999 9 1106 15 10.1101/gr.9.11.1106 10568750 PMC310826

[B22] Hosack DA Dennis G Sherman BT Lane HC Lempicki RA Identifying biological themes within lists of genes with EASE Genome Biology 2003 4 P4 10.1186/gb-2003-4-6-p4 PMC328459 14519205

[B23] Ingenuity systems http://www.ingenuity.com/

