# Supplementary figures and images for: Genome-wide transcription factor binding site/promoter databases for the analysis of gene sets and co-occurrence of transcription factor binding motifs
Source: BMC Genomics. 2010 Mar 1;11:145. doi: 10.1186/1471-2164-11-145 (PMC2841680; doi:10.1186/1471-2164-11-145)

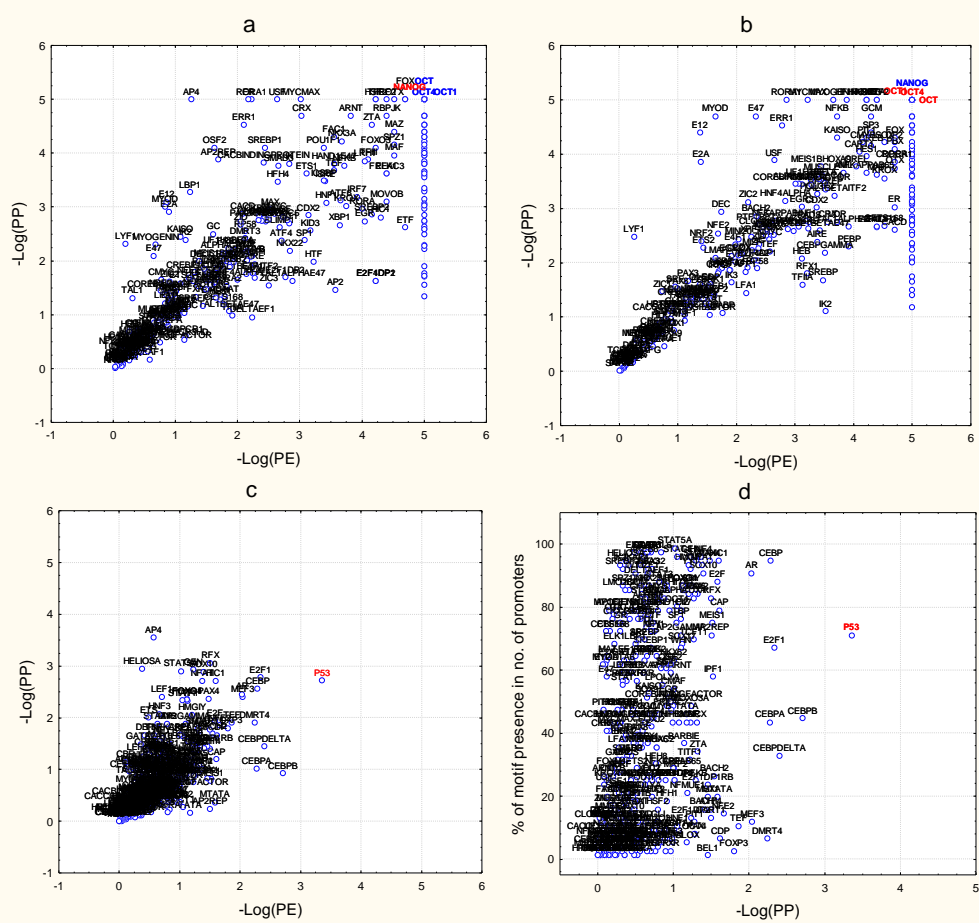

Supplement: Additional file 1 — Figure S1. Promoter analysis of genes binding NANOG, OCT4, and TP53 as determined by ChIP-Chip analyses. All results based on 105 re-samples for estimations of PE and PP a) analysis of promoters of NANOG binding genes using phylogenic foot printing of the -5000,+1000 portions of the promoters. b) Analysis of promoters of OCT4 binding genes using phylogenic foot printing of the -5000,+1000 portions of the promoters. All TBFS names having PE values equal to 5 except NANOG, OCT, OCT1, and OCT4 omitted from the graph for clarity. c) Analysis of promoters of TP53 binding genes using the -1500,+500 portions of the promoters. d) Analysis of promoters of TP53 binding genes using phylogenic foot printing of the -5000,+1000 portions of the promoters. [file 1471-2164-11-145-S1.PDF]

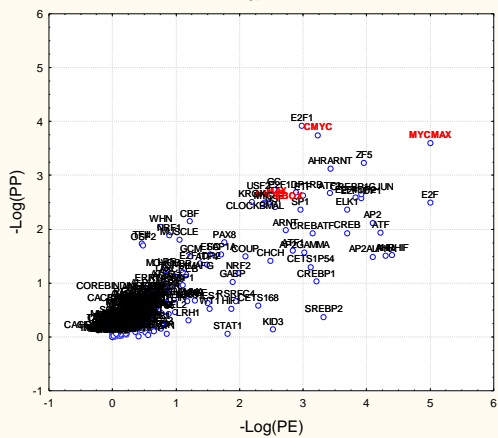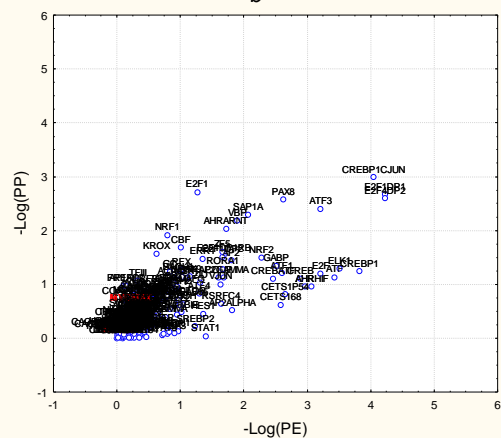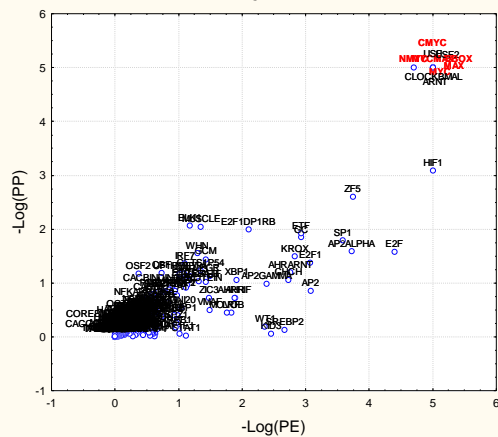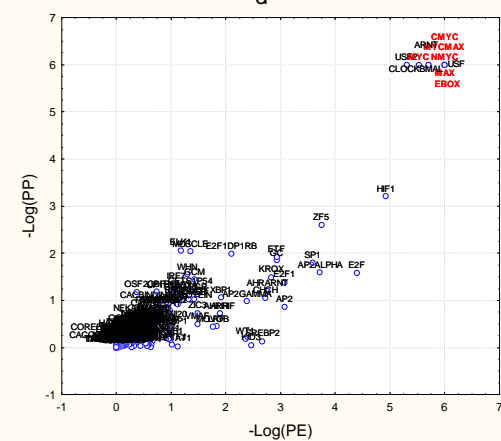

Supplement: Additional file 2 — Figure S2. Promoter analysis of genes binding MYC as determined by ChIP-Chip analysis. a) Analysis of promoters of all MYC binding genes in the ZELLER_MYC data. b) Analysis of promoters of MYC binding genes lacking EBOXes c) Analysis of promoters of MYC binding genes with EBOXes. d) Analysis of promoters of MYC binding genes with EBOXes using 106 instead of 105 re-samples to compute PE and PP. [file 1471-2164-11-145-S2.PDF]

a

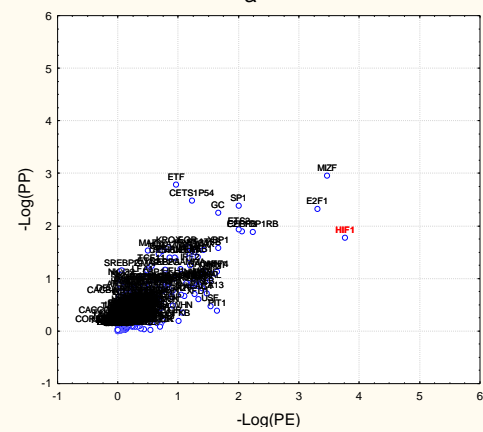

b

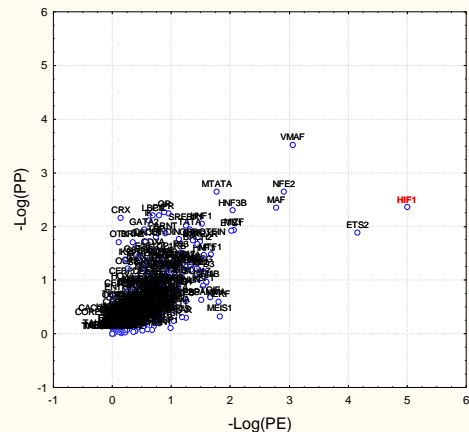

c

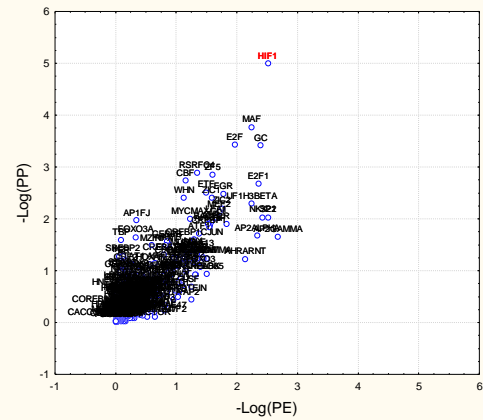

d

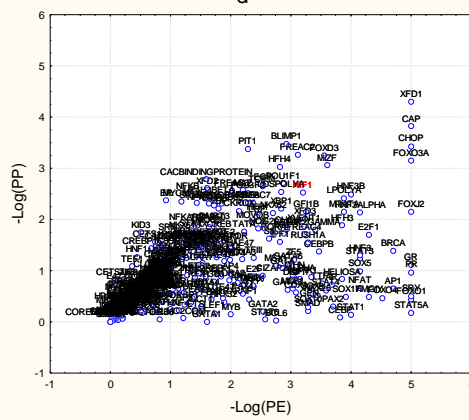

Supplement: Additional file 4 — Figure S4. Promoter analysis of hypoxia related gene signatures obtained from MSigDB, using phylogenic foot printing and 105 re-samples for estimations of PE and PP. a) Results for the MENSE_HYPOXIA_UP signature. b) Results for the HIF_TARGETS signature. c) Results for the HYPOXIA_NORMAL_UP signature. d) Results for the MANALO_HYPOXIA_UP signature. All signature designations are as in MSigDB. [file 1471-2164-11-145-S4.PDF]

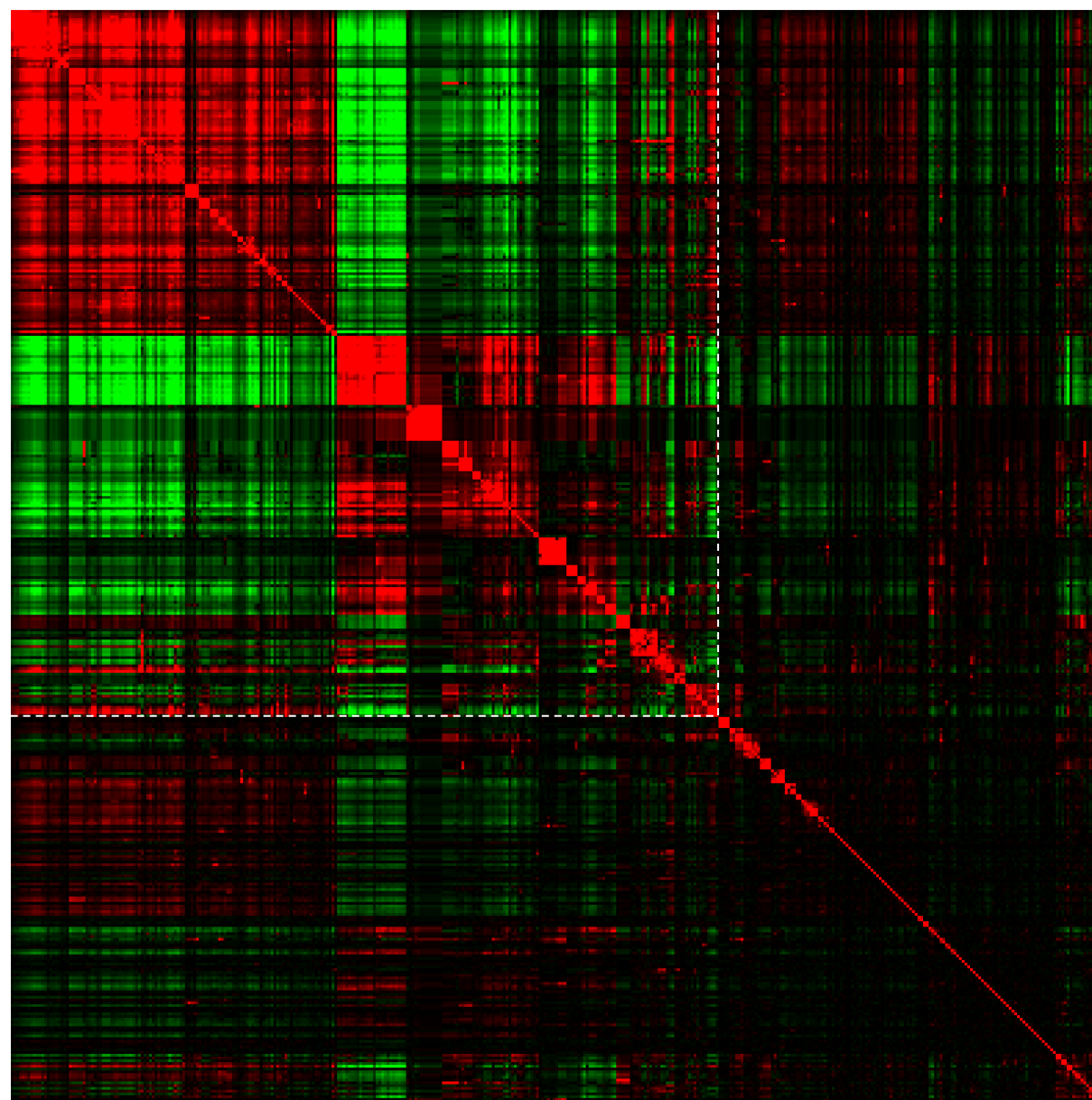

Supplement: Additional file 5 — Figure S5. Analysis of correlations among TFBS obtained using the -1500,+500 portions of the promoters. Heat map obtained after reorganization with hierarchical cluster analysis. The portion of TFBS used for further analyses in box. [file 1471-2164-11-145-S5.PDF]

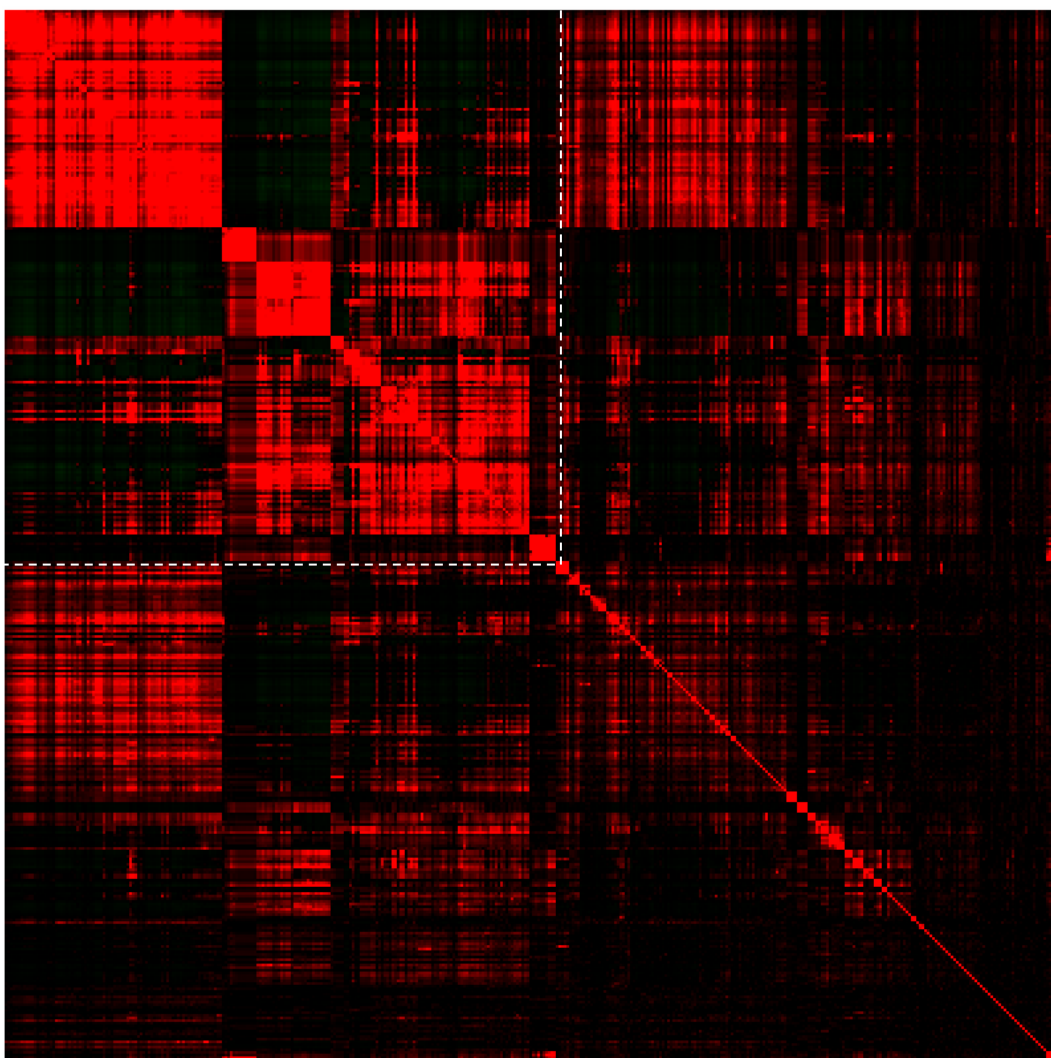

Supplement: Additional file 7 — Figure S6. Analysis of correlations among TFBS obtained using the -5000,+1000 portions of the promoters and phylogenic foot printing. Heat map obtained after reorganization with hierarchical cluster analysis. The portion of TFBS used for further analyses in box. [file 1471-2164-11-145-S7.PDF]
