# Supplementary material for: Genome-wide transcription factor binding site/promoter databases for the analysis of gene sets and co-occurrence of transcription factor binding motifs
Source: BMC Genomics. 2010 Mar 1;11:145. doi: 10.1186/1471-2164-11-145 (PMC2841680; doi:10.1186/1471-2164-11-145)
Supplement: Additional file 3 — Figure S3. Promoter analysis of MYC gene signatures obtained from MSigDB, using the -5000,+1000 portions of the promoters, phylogenic foot printing, and 105 re-samples for estimations of PE and PP. a) Results for the FERNANDEZ_MYC_TARGETS signature. b) Results for the MYC_ONCOGENE_SIGNATURE signature. c) Results for the MYC_TARGETS signature. d) Results for the LEE_MYC_UP signature. All signature designations are as in MSigDB. [file 1471-2164-11-145-S3.PDF]

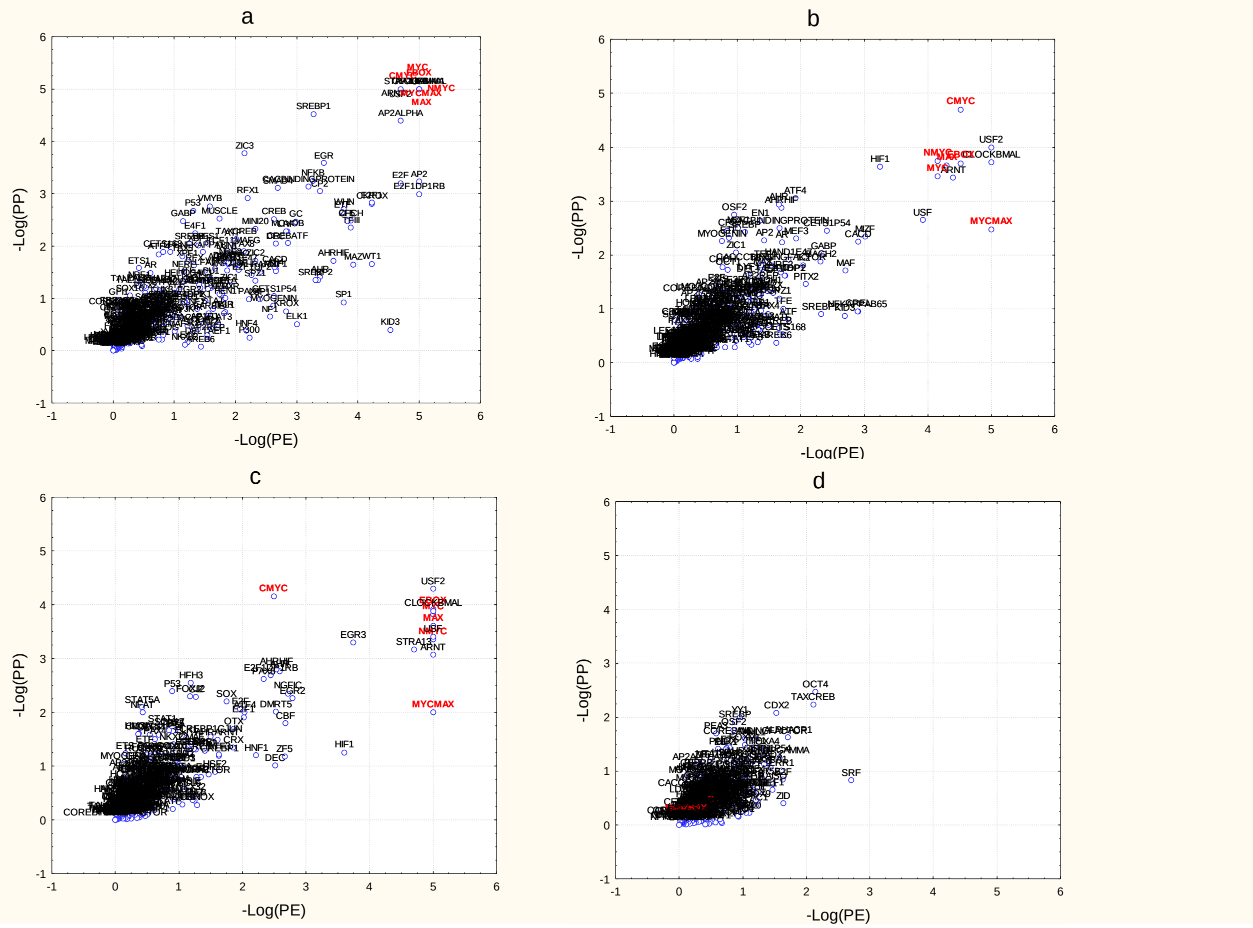

a
b
c
d
-Log(PP)
-Log(PE)
CMYC
MYCMAX
USF2
SREBP1
ZIC3
HIF1
OCT4
TAXCREB
SRF
